# Formation of Mo_5_Si_3_/Mo_3_Si–MgAl_2_O_4_ Composites via Self-Propagating High-Temperature Synthesis

**DOI:** 10.3390/molecules25010083

**Published:** 2019-12-24

**Authors:** Chun-Liang Yeh, Yin-Chien Chen

**Affiliations:** Department of Aerospace and Systems Engineering, Feng Chia University, Taichung 40724, Taiwan; m0626603@fcu.edu.tw

**Keywords:** intermetallic/ceramic composite, self-propagating high-temperature synthesis, Mo_5_Si_3_, Mo_3_Si, MgAl_2_O_4_

## Abstract

In situ formation of intermetallic/ceramic composites composed of molybdenum silicides (Mo_5_Si_3_ and Mo_3_Si) and magnesium aluminate spinel (MgAl_2_O_4_) was conducted by combustion synthesis with reducing stages in the mode of self-propagating high-temperature synthesis (SHS). The SHS process combined intermetallic combustion between Mo and Si with metallothermic reduction of MoO_3_ by Al in the presence of MgO. Experimental evidence showed that combustion velocity and temperature decreased with increasing molar content of Mo_5_Si_3_ and Mo_3_Si, and therefore, the flammability limit determined for the reaction at Mo_5_Si_3_ or Mo_3_Si/MgAl_2_O_4_ = 2.0. Based upon combustion wave kinetics, the activation energies, *E*_a_ = 68.8 and 63.8 kJ/mol, were deduced for the solid-state SHS reactions producing Mo_5_Si_3_– and Mo_3_Si–MgAl_2_O_4_ composites, respectively. Phase conversion was almost complete after combustion, with the exception of trivial unreacted Mo existing in both composites and a minor amount of Mo_3_Si in the Mo_5_Si_3_–MgAl_2_O_4_ composite. Both composites display a dense morphology formed by connecting MgAl_2_O_4_ crystals, within which micro-sized molybdenum silicide grains were embedded. For equimolar Mo_5_Si_3_– and Mo_3_Si–MgAl_2_O_4_ composites, the hardness and fracture toughness are 14.6 GPa and 6.28 MPa m^1/2^, and 13.9 GPa and 5.98 MPa m^1/2^, respectively.

## 1. Introduction

Transition metal silicides are considered as a unique class of intermetallic compounds for ultra-high-temperature applications. In the Mo–Si binary system, there exist three silicides—MoSi_2_, Mo_5_Si_3_, and Mo_3_Si. Among them, Mo_5_Si_3_ with a melting point of 2180 °C is the most refractory and MoSi_2_ is the most studied phase. MoSi_2_ has relatively low density, excellent oxidation and corrosion resistance, high thermal and electric conductivities, and good compatibility with many ceramic reinforcements [1,2]. Recently, Mo_5_Si_3_ has attracted growing interests not only due to its high melting point, but also because of its large alloying potential, wide homogeneity range, high strength, and creep resistance superior to MoSi_2_ [3,4]. Mo_3_Si is an important structural material as well, especially in the development of α-Mo–Mo_5_SiB_2_–Mo_3_Si composites [5,6]. Because silicides and ceramics are two sorts of high-temperature materials highly synergistic with each other, mechanical properties of silicide intermetallics can be effectively improved by the addition of ceramic phases [7,8,9].

Magnesium aluminate spinel (MgAl_2_O_4_) possesses many advantageous properties, including high hardness (16.1 GPa), high melting point (2135 °C), relatively low density (3.58 g/cm^3^), high chemical inertness, low thermal expansion coefficient, high mechanical strength, and good thermal shock resistance [10,11]. However, both conventional solid-state reaction and wet chemical methods require lengthy processing time and complicated synthesis steps to obtain MgAl_2_O_4_ with specific characteristics [12,13,14].

Because ceramic-added MoSi_2_ has been extensively investigated, this study aims to prepare molybdenum silicides (Mo_5_Si_3_ and Mo_3_Si) and MgAl_2_O_4_ composites by a facile fabrication route involving solid-phase combustion reaction with metallothermic reduction. The synthesis reaction was formulated with sufficient energy release to proceed in the self-propagating high-temperature synthesis (SHS) mode. The SHS process has the merits of high energy effectiveness, short reaction time, simple operation, and good purification capability [15,16,17]. Aluminothermic reduction of metal oxides is thermally beneficial for combustion synthesis of materials with low formation enthalpies and provides in situ generation of Al_2_O_3_ [18,19,20]. The SHS reaction combined with aluminothermic reduction was conducted with green compacts made up of MoO_3_, SiO_2_, Al, and MgO for the synthesis of MoSi_2_– and Mo_5_Si_3_–MgAl_2_O_4_ composites [21]. According to Zaki et al. [21], it required excess MoO_3_ to compensate for its volatilization loss and a higher reaction pressure to suppress the vaporization of MoO_3_. Horvitz and Gotman [22] performed combustion synthesis in both SHS and thermal explosion modes using powder compacts of 2TiO_2_–Mg–4Al to fabricate Al_2_O_3_-rich spinel and two aluminides—TiAl and Ti_3_Al. Recently, Omran et al. [23] studied reduction of WO_3_ and B_2_O_3_ by Mg in the presence of Al_2_O_3_ to prepare MgAl_2_O_4_–W and MgAl_2_O_4_–W–W_2_B composites in the SHS manner. As proposed by Omran et al. [23], MgAl_2_O_4_ was formed through dissolution of MgO into the Al_2_O_3_ melt.

On in situ formation of MgAl_2_O_4_ and Mo_5_Si_3_ or Mo_3_Si composites, this study explores the influence of the stoichiometry of reactants on the flammability limit and combustion wave kinetics of the SHS reaction. The phase composition and microstructure of the final products were characterized and the hardness and fracture toughness were examined.

## 2. Materials and Methods

The starting materials included MoO_3_ (Acros Organics, 99.5%, Waltham, MA, USA), Mo (Strem Chemicals, <45 µm, 99.9%, Newburyport, MA, USA), Al (Showa Chemical Co., <45 µm, 99.9%, Tokyo, Japan), Si (Strem Chemicals, <45 µm, 99.5%), and MgO (Alfa Aesar, 99%, Ward Hill, MA, USA). Two reaction systems, formulated below, were composed of the Al–MoO_3_ thermite and Mo, Si, and MgO for the synthesis of Mo_5_Si_3_– and Mo_3_Si–MgAl_2_O_4_ composites.

(1) $$MoO_{3}+2Al+(5x-1)Mo+3xSi+MgO\to xMo_{5}Si_{3}+MgAl_{2}O_{4}$$

(2) $$MoO_{3}+2Al+(3y-1)Mo+ySi+MgO\to yMo_{3}Si+MgAl_{2}O_{4}$$

where stoichiometric coefficients, *x* and *y*, in Reactions (1) and (2) signify Mo_5_Si_3_ and Mo_3_Si produced per unit mole of MgAl_2_O_4_ in the composites.

Within two thermite-containing SHS systems, the MoO_3_ + 2Al reaction is extremely energetic with the reaction heat of Δ*H* = −930.7 kJ and an adiabatic temperature of *T_ad_* = 4280 K [24]. However, formation enthalpies of Mo_5_Si_3_ and Mo_3_Si (i.e., Δ*H_f_* = −310.6 and −118.4 kJ/mol [25]) are much lower than the heat released from the MoO_3_ + 2Al thermite. The addition of Mo, Si, and MgO has a dilution effect on the combustion reaction. Moreover, the adiabatic temperatures of intermetallic reactions between Mo and Si to form Mo_5_Si_3_ and Mo_3_Si are lower than the criterion temperature (1800 K) required for self-sustaining combustion [15]. Therefore, it is believed that in the reaction mechanisms, aluminothermic reduction of MoO_3_ is the initiation step.

The adiabatic combustion temperatures (*T_ad_*) associated with Reactions (1) and (2) under different stoichiometric coefficients were calculated according to the following equation [21,26] with thermochemical data taken from [25]:

(3) $$\Delta H_{r}+\int_{298}^{T_{ad}}\sum n_{j}C_{p}(P_{j})dT+\sum_{298-T_{ad}}n_{j}L(P_{j})=0$$

where Δ*H_r_* is the reaction enthalpy at 298 K, *n_j_* is the stoichiometric constant, *C_p_* and *L* are the heat capacity and latent heat, and *P_j_* refers to the product.

The SHS experiments were conducted in a combustion chamber equipped with quartz viewing windows and filled with high-purity (99.99%) argon. The reactant powders were mixed in a ball mill. Then, the powder mixture was uniaxially compressed in a stainless-steel mold at a pressure of 60–70 MPa to form cylindrical test specimens 7 mm in diameter, 12 mm in height, and with a relative density of 60%. The propagation velocity of combustion wave (*V*_f_) was determined from the time series of recorded combustion images. The reaction temperature was measured by a bare wire Pt/Pt–13%Rh thermocouple with a bead diameter of 125 µm. In this study, a two-hole ceramic protection tube was used to accommodate the fine wire thermocouple. The junction was exposed outside the protection tube and the length of the wire protruding from the tube was only about 3 mm. Because the ceramic tube was well mounted and the protruding length was short, the whole setup was rigid and the thermocouple bead had good contact with the sample. Details of the experimental scheme were described elsewhere [27].

According to Bradley and Matthews [28], the conduction loss from the thermocouple depends on the length of wire between the junction and the support. The thermocouple used in this study was 40 mm in length and 62.5 µm in wire diameter. This dimension justifies the measurement practically unaffected by the conduction cooling [28]. However, the radiation loss from the thermocouple bead is another source of error in the thermocouple measurement. An estimate of the radiation correction can be obtained by assuming that a steady state exists between convective heat transfer to and radiation loss from the thermocouple bead [29].

(4) $$\Delta T=\frac{\sigma \cdot \varepsilon \cdot D}{Nu\cdot k}\left(T_{c}^{4}-T_{\infty }^{4}\right)$$

where *σ* is the Stefan–Boltzmann constant, *ε* the thermocouple emissivity, *D* the thermocouple bead diameter, *Nu* the Nusselt number, and *k* the gas thermal conductivity.

Product constituents were analyzed by an X-ray diffractometer (Bruker D2, Billerica, MA, USA) using CuKα radiation. Examination of the microstructure and elemental proportion of the composite was performed by a scanning electron microscope (Hitachi S3000H, Tokyo, Japan) and energy dispersive spectroscopy (EDS). Moreover, Vickers hardness (*H*) and fracture toughness (*K*_IC_) of the final products were measured [30,31]. For the hardness and fracture toughness measurement, the selected experiments of Reaction (1) with *x* = 1.0 and Reaction (2) with *y* = 1.0 were conducted by placing the powder compact in a steel mold. Densification of the product was accomplished by a hydraulic press machine. Upon completion of the self-sustaining combustion reaction, the burned sample was quickly pressed when the product was still hot and plastic, which was held for about 15 s. The density of the pressed products reached about 98% of theoretical density. The surface of the specimens was then polished for the measurement.

## 3. Results and Discussion

### 3.1. Combustion Wave Kinetics and Activation Energy

**Figure 1:**
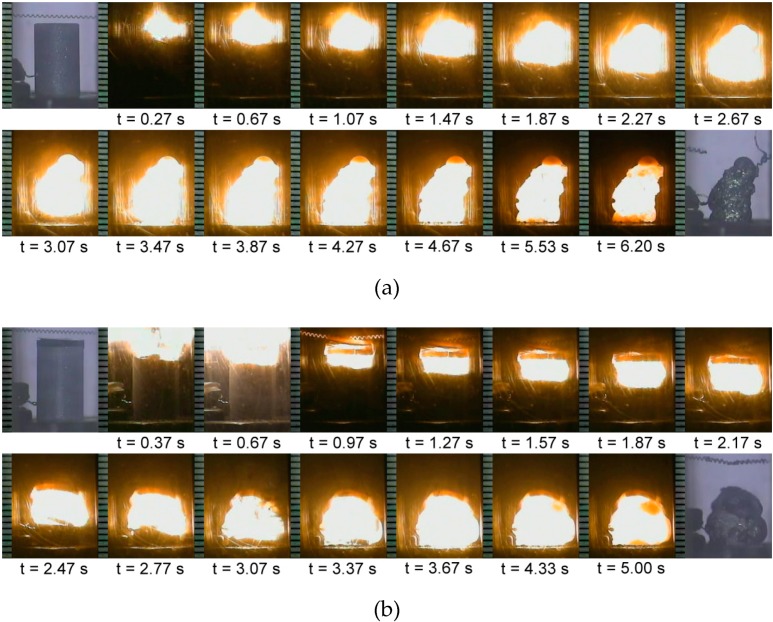
Self-propagating combustion images recorded from (**a**) Reaction (1) with *x* = 1.5 and (**b**) Reaction (2) with *y* = 1.75.

Figure 1a,b presents typical sequences of combustion pictures recorded from Reactions (1) and (2), respectively, and shows that upon ignition, self-sustaining combustion is established. With the progression of combustion wave, as illustrated in Figure 1, the reacted samples are partially molten and shrunk. Sample shrinkage during the SHS process was due perhaps to the low melting points of thermite reagents (795 °C for MoO_3_ and 660 °C for Al). The presence of liquid melt contributed to self-densification of the final product. As mentioned above, reaction exothermicity tends to weaken with increasing molar content of molybdenum silicides. Based on experimental evidence, the flammability limits of Reactions (1) and (2) in terms of their stoichiometric coefficients exist at *x* = 2.0 and *y* = 2.0, respectively. That is, combustion ceased to propagate and quenched as long as the stoichiometric coefficient of the reaction exceeded its flammability limit. Nevertheless, for the powder compacts with Mo and Si lesser than a critical amount (e.g., *x* < 1.0 and *y* < 1.0), combustion was violent and occurred almost in a manner of thermal explosion. This caused massive sample melting and difficulty in characterization of the combustion wave. Therefore, the scope of experimental variables conducted by this study was set stoichiometric coefficients (*x* and *y*) between 1.0 and 2.0.

**Figure 2:**
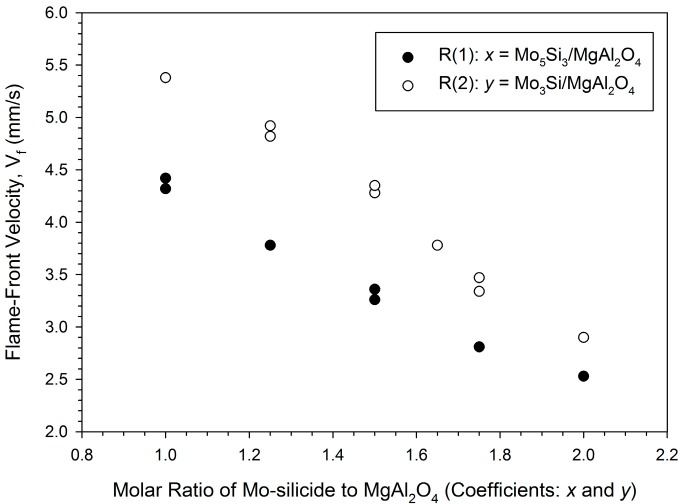
Effect of molar ratio of Mo-silicide to MgAl_2_O_4_ on flame-front velocities of Reactions (1) and (2).

Figure 2 shows a decrease in the combustion wave velocity with increasing silicide content for both reaction systems. The combustion wave velocity roughly ranges between 2.5 and 5.4 mm/s. As stated earlier, the increase of Si and Mo powders in the samples has a cooling effect on combustion. Formation of Mo_5_Si_3_ in Reaction (1) requires larger amounts of Mo and Si than the synthesis of Mo_3_Si in Reaction (2). Consequently, at equal stoichiometric coefficients, Reaction (2) shows a higher combustion front velocity than Reaction (1).

**Figure 3:**
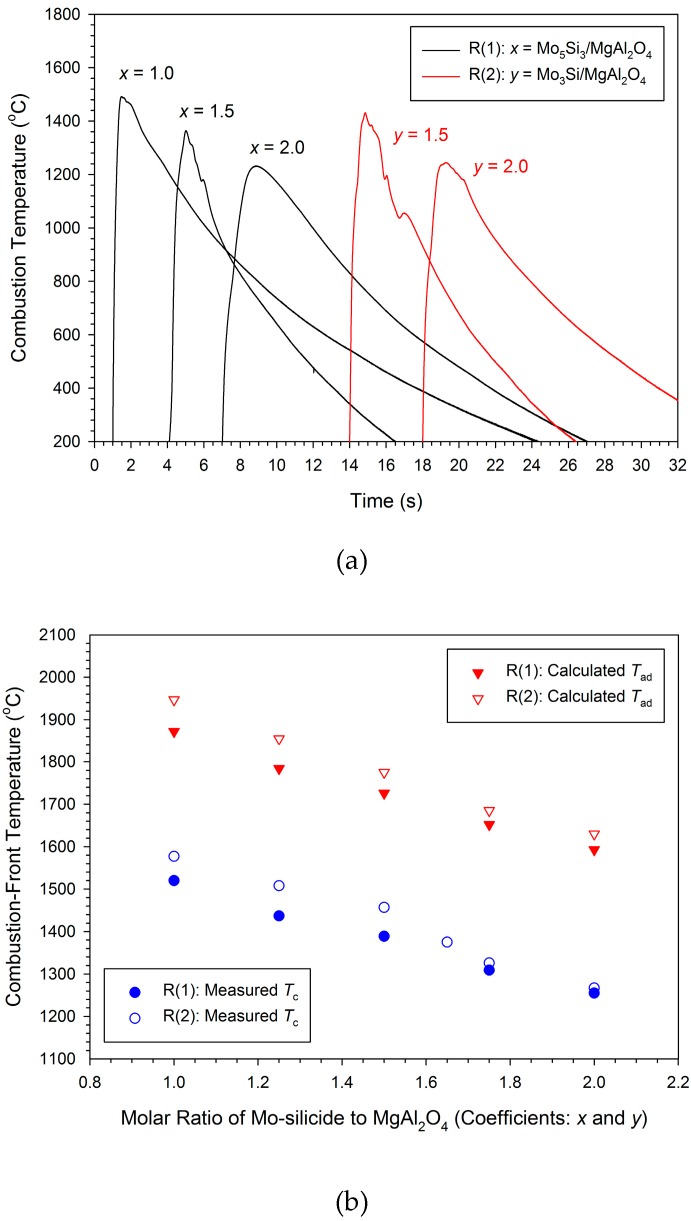
Effect of molar ratio of Mo-silicide to MgAl_2_O_4_ on combustion temperatures of Reactions (1) and (2): (**a**) Typical temperature profiles and (**b**) combustion front temperatures.

Typical combustion temperature profiles presenting the reaction exothermicity of different powder compacts are plotted in Figure 3a. The temperature profiles feature a sudden rise, signifying a rapid arrival of the combustion wave, and the pinnacle representing the combustion front temperature (*T*_c_). A substantial temperature decline after the passage of combustion wave is a result of heat loss to the surroundings. Based on the estimation of Equation (4), the radiation correction for the measured combustion front temperatures is in the range of 23–28 °C, which is more significant in the regions of higher temperatures. Figure 3b shows the variation of combustion front temperature with stoichiometric coefficients of Reactions (1) and (2). As indicated in Figure 3b, for Reaction (1), the increase of *x* from 1.0 to 2.0 lowers the measured combustion front temperature from 1520 to 1255 °C, which confirms the dilution effect on combustion by increasing Si and Mo. Similar decline of the reaction zone temperature was observed for Reaction (2), which shows a decrease of *T*_c_ from 1577 to 1267 °C with an increase of *y* from 1.0 to 2.0. It should be noted that at equal stoichiometric coefficients, the combustion front temperature of Reaction (2) is higher than that of Reaction (1).

The calculated adiabatic combustion temperatures are also presented in Figure 3b and signify a decline with increasing stoichiometric coefficients for both reaction systems. The values of *T_ad_* decrease from 1872 to 1593 °C for Reaction (1) and 1947 to 1630 °C for Reaction (2). This means that the exothemicity of Reaction (2) is higher than that of Reaction (1). Although the MoO_3_ + 2Al reaction is highly energetic, the synthesis of Mo_5_Si_3_ and Mo_3_Si from solid-phase combustion is weakly exothermic. According to Merzhanov [32], the adiabatic combustion temperatures associated with formation of Mo_5_Si_3_ and Mo_3_Si are as low as 1457 and 1547 °C, respectively. Moreover, the heat capacity of MgAl_2_O_4_ is larger than Al_2_O_3_ [25], and pre-added MgO acts as a diluent of combustion. That is, the production of MgAl_2_O_4_ and Mo_5_Si_3_ or Mo_3_Si imposed a cooling effect on the MoO_3_ + 2Al reaction.

When compared with the measured combustion front temperatures reported in Figure 3b, the calculated values are higher by about 350 °C. It is believed that during the SHS process, the burning sample experienced substantial heat losses to the surrounding argon gas by conduction, by convection, or by both, and to the chamber wall by radiation, which resulted in the actual combustion temperature lower than the adiabatic value.

**Figure 4:**
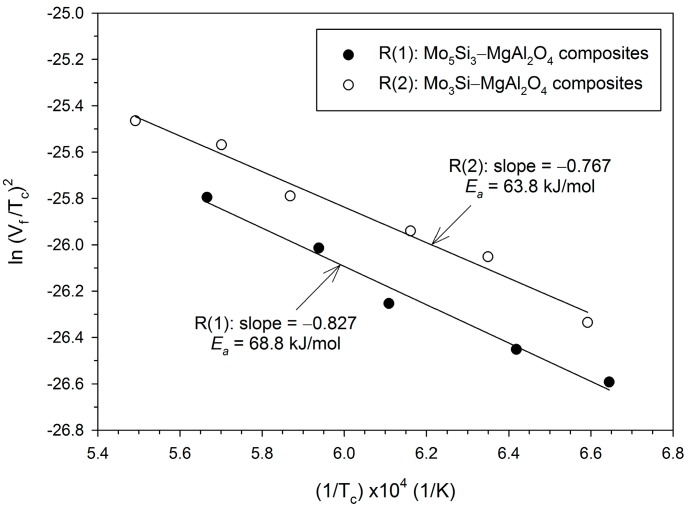
Correlation for determination of activation energies (*E*_a_) for formation of Mo_5_Si_3_– and Mo_3_Si–MgAl_2_O_4_ composites from solid-phase combustion synthesis.

According to combustion wave kinetics [33,34], the activation energy (*E*_a_) of solid-phase combustion can be deduced from the correlation between ln(*V*_f_/*T*_c_)^2^ and 1/*T*_c_ in a form of linear relationship. Based on Arrhenius kinetics of the solid-state reaction, the activation energy is dependent on the reaction mechanism. Figure 4 plots the correlation of two sets of experimental data with their individual best-fitted linear lines. From the slopes of the linear lines, *E*_a_ = 68.8 and 63.8 kJ/mol were deduced for Reactions (1) and (2), respectively. It can be seen that two activation energies are close to each other. This implies that both reactions are governed by similar reaction mechanisms, which involve aluminothermic reduction of MoO_3_ as the initiation step, followed by a combination of Al_2_O_3_ and MgO to form MgAl_2_O_4_, and intermetallic interactions between Mo and Si to produce silicide compounds. The increase of Si and Mo in the sample reduces the combustion exothermicity, but produces no change in the reaction mechanisms. Compared with *E*_a_ = 54 kJ/mol for the Al–MoO_3_ thermite reaction [35], the synthesis reactions of this study have a higher kinetic barrier. This could most likely be caused by the fact that for Reactions (1) and (2), intermetallic and combination reactions need to continue to form Mo_5_Si_3_ or Mo_3_Si and MgAl_2_O_4_ after reduction of MoO_3_ by Al. It should be noted that the actual reaction mechanism might be more complex than that proposed above, because the proceeding reactions could occur in both a sequential and parallel manner during combustion synthesis.

### 3.2. Microstructure and Composition of SHS-Derived Composites

**Figure 5:**
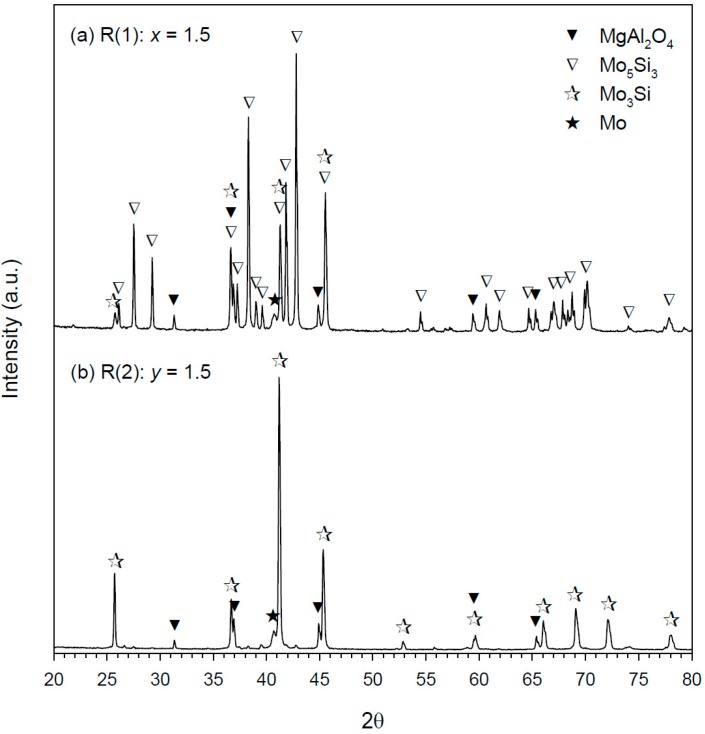
X-ray diffraction (XRD) patterns of Mo_5_Si_3_–MgAl_2_O_4_ and Mo_3_Si–MgAl_2_O_4_ composites synthesized from (**a**) R(1): *x* = 1.5 and (**b**) R(2): *y* = 1.5.

Phase constituents of the SHS-derived composites from Reaction (1) with *x* = 1.5 and Reaction (2) with *y* = 1.5 are unveiled in Figure 5a,b, respectively. As shown in Figure 5a, in addition to Mo_5_Si_3_ and MgAl_2_O_4_ as major phases, there exist two minor species Mo_3_Si and Mo. The presence of Mo_3_Si could be caused by the interaction of Mo with Mo_5_Si_3_ via 4Mo + Mo_5_Si_3_ → 3Mo_3_Si [7]. A small amount of remnant Mo is possibly because Mo_5_Si_3_ has a homogeneity range from 37.5 to 40% Si [36]. On the other hand, Mo_3_Si is a line compound. Figure 5b shows near completion of phase conversion for Reaction (2). The as-synthesized product is a Mo_3_Si–MgAl_2_O_4_ composite with a trivial amount of Mo.

**Figure 6:**
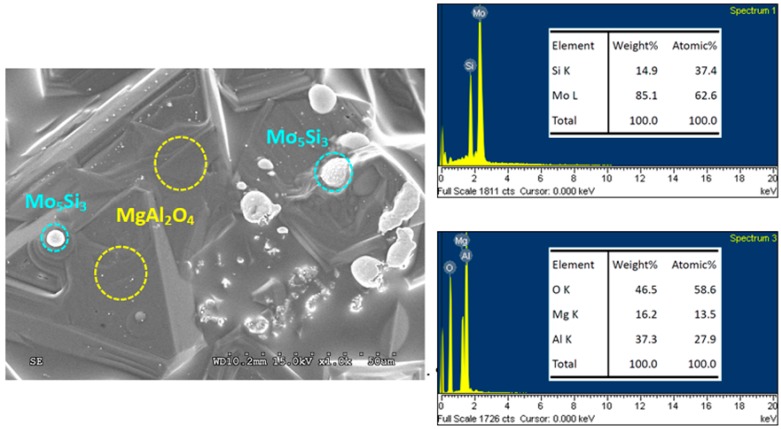
Scanning electron microscopy (SEM) micrograph and energy dispersive spectroscopy (EDS) spectra of Mo_5_Si_3_/MgAl_2_O_4_ composite obtained from Reaction (1) with *x* = 1.5.

**Figure 7:**
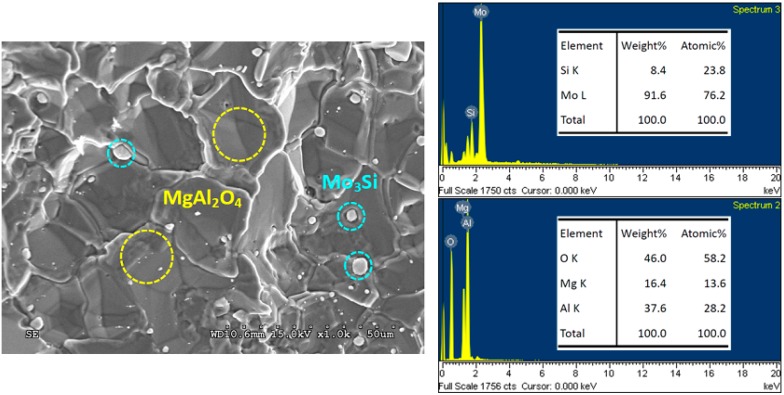
SEM micrograph and EDS spectra of Mo_3_Si/MgAl_2_O_4_ composite obtained from Reaction (2) with *y* = 1.5.

Scanning electron microscopy (SEM) micrographs in Figure 6; Figure 7 illustrate the microstructure of fracture surface of Mo_5_Si_3_– and Mo_3_Si–MgAl_2_O_4_ composites obtained from Reactions (1) and (2), respectively. The composites display a dense and connecting morphology with embedded granular silicide particles. It is believed that the presence of liquid melt in the course of the SHS process aided in densification of the final product. As revealed in Figure 6, MgAl_2_O_4_ crystals form a dense matrix and Mo_5_Si_3_ grains are embedded in MgAl_2_O_4_ or distributed over the surface. The particle size of Mo_5_Si_3_ ranges from 2 to 10 µm. The minor silicide phase Mo_3_Si formed from Reaction (1) is not shown in Figure 6. Because most of the silicide grains were embedded in the composite and only a small amount of them were present on the fracture surface of the sample, the minor phase was sometimes difficult to find on a small area of the sample. On the other hand, the X-ray diffraction (XRD) analysis was performed with the whole specimen. Therefore, all components were identified. A similar microstructure is observed in Figure 7, showing that large MgAl_2_O_4_ crystals are connected and small Mo_3_Si grains formed from Reaction (2) have a particle size around 2–5 µm.

EDS spectra and associated element ratios for MgAl_2_O_4_, Mo_5_Si_3_, and Mo_3_Si are also presented. In Figure 6, large connecting crystals with Mg:Al:O = 13.5:27.9:58.6 are certainly MgAl_2_O_4_ and small grains, and Mo:Si = 62.6:37.4 are Mo_5_Si_3_. In Figure 7, MgAl_2_O_4_ and Mo_3_Si phases are determined to have Mg:Al:O = 13.6:28.2:58.2 and Mo:Si = 76.2:23.8, respectively. EDS analysis confirms that atomic ratios of the compounds are very close to their stoichiometries.

On account of sample melting during the SHS process, the relative density of as-synthesized composites measured by the Archimedes method reached up to 90–94%. Vickers hardness of 14.6 GPa and fracture toughness of 6.28 MPa m^1/2^ were determined for an equimolar Mo_5_Si_3_–MgAl_2_O_4_ composite. Compared with pure Mo_5_Si_3_ (*H* = 13.1 GPa and *K*_IC_ = 2.37 MPa m^1/2^) [7], the mechanical properties of Mo_5_Si_3_–MgAl_2_O_4_ composite are improved. Similarly, the equimolar Mo_3_Si–MgAl_2_O_4_ composite possesses higher hardness of 13.9 GPa and fracture toughness of 5.98 MPa m^1/2^, in comparison to single phase Mo_3_Si (*H* = 13 GPa and *K*_IC_ = 2–3 MPa m^1/2^) [37].

## 4. Conclusions

In situ formation of Mo_5_Si_3_– and Mo_3_Si–MgAl_2_O_4_ composites was investigated by combustion synthesis in the SHS mode. The reaction mechanisms involve metallothermic reduction of MoO_3_ with Al, followed by intermetallic interactions of Si with Mo and a combination reaction of Al_2_O_3_ with MgO. Activation energies of the solid-phase SHS reactions, *E*_a_ = 68.8, and 63.8 kJ/mol, were deduced from the correlation of combustion wave velocity with reaction temperature for formation of Mo_5_Si_3_– and Mo_3_Si–MgAl_2_O_4_ composites, respectively. The increase of silicide molar content in the composite decreased the reaction exothermicity and led to flammability limits for combustion synthesis of the composites at molar ratios of Mo_5_Si_3_/MgAl_2_O_4_ = 2.0 and Mo_3_Si/MgAl_2_O_4_ = 2.0. Phase conversion from the reactants to final products was almost complete, except for small quantities of Mo and Mo_3_Si remaining in the Mo_5_Si_3_–MgAl_2_O_4_ composite and a negligible amount of Mo in the Mo_3_Si–MgAl_2_O_4_ composite. Dense products with relative density of 90–94% were obtained. Mo_5_Si_3_ and Mo_3_Si grains were embedded in the continuous MgAl_2_O_4_ crystals. Vickers hardness of 14.6 and 13.9 GPa and fracture toughness of 6.28 and 5.98 MPa m^1/2^ were determined for Mo_5_Si_3_– and Mo_3_Si–MgAl_2_O_4_ composites, respectively. The addition of MgAl_2_O_4_ was shown to improve the mechanical properties of Mo_5_Si_3_ and Mo_3_Si. In practice, this study demonstrated an effective fabrication route for in situ formation of Mo_5_Si_3_– and Mo_3_Si–MgAl_2_O_4_ composites.
